# 571. Development of a Multiplexed PCR Assay for the Detection and Differentiation of Non-Tuberculosis Mycobacteria (NTM)

**DOI:** 10.1093/ofid/ofad500.640

**Published:** 2023-11-27

**Authors:** Scott Cowden, James Grantham, Jerod Davidson, Emily Lute, Jamie Nutt, Ellis Bixler, Steve Kleiboecker

**Affiliations:** Eurofins Viracor Clinical Diagnostics, Lenexa, Kansas; Eurofins Viracor, Lenexa, Kansas; Eurofins Viracor, Lenexa, Kansas; Eurofins Viracor, Lenexa, Kansas; Eurofins Viracor, Lenexa, Kansas; Eurofins-Viracor, Lenexa, Kansas; Eurofins-Viracor, Lenexa, Kansas

## Abstract

**Background:**

Current NTM diagnostics rely on a combination of testing modalities including clinical presentation, radiology, and culture. NTM cultures often have extended turnaround times. Additionally, the importance of discriminating etiological agents in the *Mycobacterium avium* complex (MAC) and *Mycobacterium abscessus* complex (MAbC) due to increasing drug resistance is now recognized. Here we describe the development of real-time PCR assays targeting members of the MAC and MAbC responsible for NTM infections. The objective was to develop a rapid diagnostic assay for the detection of pathogenic NTM directly from patient blood and sputum.

**Methods:**

The MAC qPCR assay design allows for non-specific yet inclusive detection of *M. avium* subsp. *avium*, *hominissuis*, *paratuberculosis*, and *sylvaticum*, *M. intracellulare*, *M. intracellulare* subsp. *chimaera* and *M. colombiense.* The MAbC assay allows for inclusive and differential detection of *M. abscessus* subsp. *abscessus*, *bolletii* and *massiliense*. MAC and MAbC designs were screened to identify candidates and were then multiplexed with an internal control assay. Performance was evaluated in spiked whole blood and sputum.

DNA was extracted using the MagMAX™ DNA Multi-Sample Ultra 2.0 Kit and KingFisher™ Flex system (Thermo Fisher). Amplification was performed using TaqMan™ Fast Advanced Master Mix (Thermo Fisher) and the Applied Biosystems™ 7500 Fast instrument.

**Results:**

Linear regression of plasmid standards for assays targeting MAbC-specific gene targets produced R^2^ values >0.99 and slopes ranging from -3.39 to -3.35 (97-99% efficiency). Linear regression of standards for assays targeting MAC-specific gene targets produced R2 values >0.99 and slopes ranging from -3.47 to -3.35 (94-98% efficiency). Assay specificity was determined by challenging the assays with NTM’s outside of each respective complex as well as other common bacterial pathogens.

**Conclusion:**

Development of NTM PCR assays to detect and differentiate slow growing *Mycobacteria* aids in rapid diagnosis and provides insight into selection of appropriate antibiotic therapies. The assays described here exhibit excellent linearity, specificity, inclusivity and with additional development will provide a reliable means of detecting and differentiating relevant NTM species.

**Disclosures:**

**Scott Cowden, MS, MB ASCP(CM)**, Eurofins Viracor: Employee **James Grantham, n/a**, Eurofins Viracor: Employee **Jerod Davidson, n/a**, Eurofins Viracor: Employee **Emily Lute, n/a**, Eurofins Viracor: Employee **Jamie Nutt, n/a**, Eurofins Viracor: Employee

